# Delayed dysphagia following foreign body swallowing: case report 

**Published:** 2021

**Authors:** Gholam Reza Mohajeri, Hamid Talebzadeh

**Affiliations:** *Department of Surgery, School of Medicine, Isfahan University of Medical Sciences, Isfahan, Iran*

**Keywords:** Swallowing, Foreign body, Dysphagia, Esophagus

## Abstract

The prevalence of foreign body swallowing is high in both children and adults. Different types of objects can be ingested. In older individuals, denture ingestion is common. The dentures may be left in the esophagus after being swallowed and then repelled. We present a case of a 62-year-old man who attended clinic due to dysphagia lasting for 5 years with an endoscopy report of a foreign body in his esophagus. There are few reports of objects remaining in the esophagus for a long time. Chronically retained foreign bodies may be associated with complications; thus, the foreign body should be promptly extracted following diagnosis, especially in the elderly.

## Introduction

 Different types of foreign objects may be swallowed by people of different ages, such as small toys, coins, keys or bones in food. Dentures are commonly ingested in older adults, and it is not uncommon for them to remain in the esophagus for a matter of time. The removal of a foreign object is often accomplished through non-surgical procedures such as endoscopy, but large or sharp-edged objects that are stuck may require surgical procedures like thoracotomy or esophagotomy. In addition, the presence of a foreign object in the esophagus is often symptomatic. Rarely does a foreign body exist in the esophagus for a long time without any symptoms. 

The presented case is a 62-year-old man who ingested his artificial teeth 9 years ago and had developed no symptoms other than dysphagia appearing 5 years ago. The patient coped with this symptom. 

## Case Reports

A 62-year-old man referred to the Department of Surgery in Isfahan with dysphagia from 5 years prior with which he had coped.

In his previous referrals to the clinic, only CXR without any contrasting means were performed and had revealed no particular points. The patient had not developed any respiratory symptoms or odynophagia. There was no report of bone swallowing in the patient's history, but endoscopic examination revealed the presence of a bone in the esophagus that had penetrated the esophageal mucosa from two sides. This investigation had been preceded by an initial endoscopy which reported an esophageal mass.

**Figure 1:**
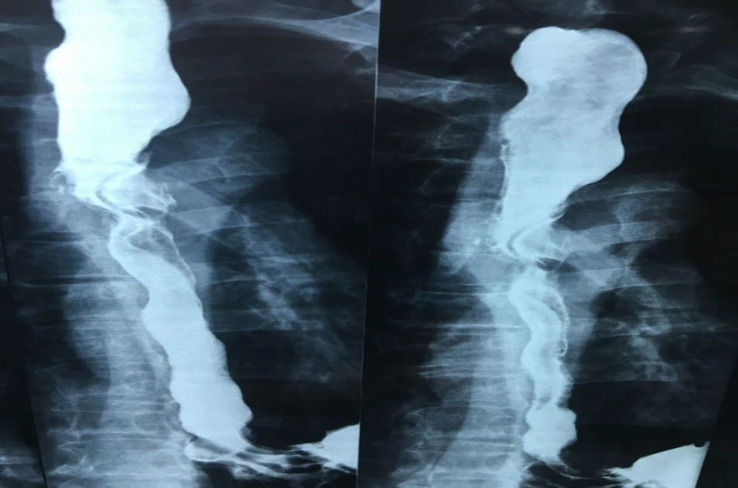
Barium swallow demonstrating proximal dilation of the esophagus following a disturbed region containing a foreign body

**Figure 2:**
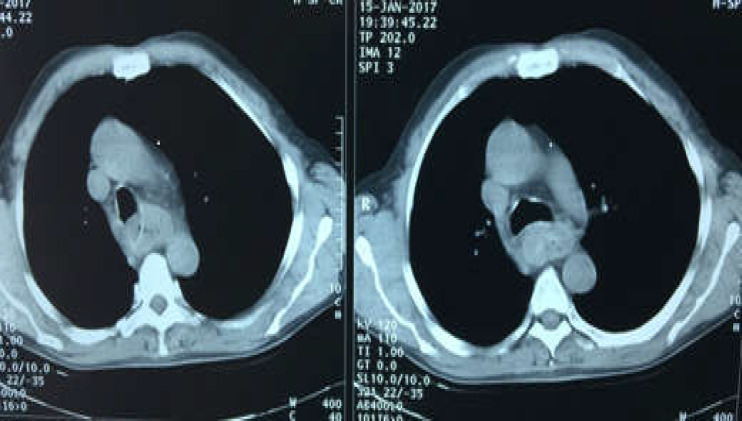
Computed tomography revealed hypodense foci in the mediastinum without definitive margins plus esophageal prominent mucosal pattern

**Figure 3:**
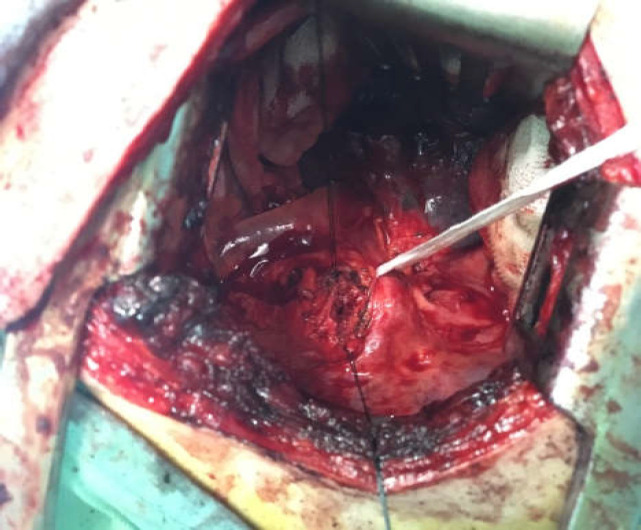
Throughout the surgery, 3 layers of the esophagus were fully restored

**Figure 4:**
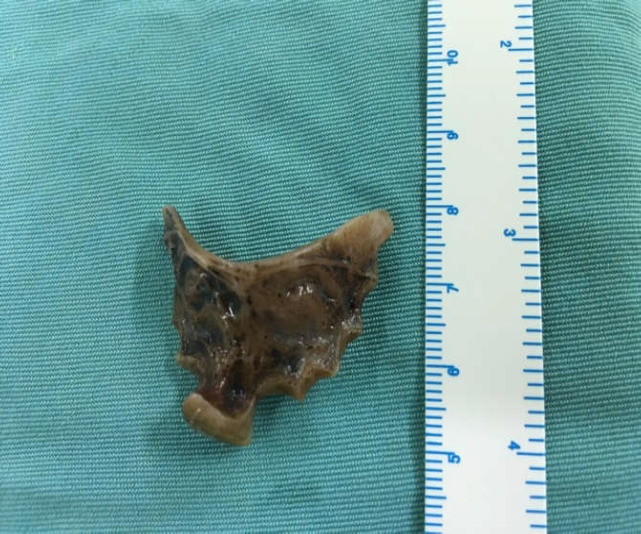
Foreign body after removal

When the patient was asked about probable foreign body ingestion, he recalled that 9 years ago he had ingested his dentures, but the patient had not been further investigated with endoscopic interventions. A barium swallow study showed dilation of the proximal part of the esophagus with a filling defect at the level of T3-T4, showing a disturbance in that region (Figure 1). Computed tomography scan (CT-scan) findings revealed the presence of a foreign body in the thoracic level of the esophagus along with widening of the proximal part of the esophagus to the hilar level (Figure 2). An esophageal prominent mucosal pattern was also reported. A further study by esophagoscopy with anesthesia demonstrated the dentures at 25 cm from the incisors. Through esophagoscopy, an attempt to withdraw the foreign body resulted in partial release of the denture, but eventually, it was fixed in the esophageal wall and extraction was not possible. Through a right thoracotomy, the foreign body was removed from the region over the arch of the azygos vein, and three layers of the esophagus were restored (Figures 3 and 4). The patient's esophagogram after 6 days revealed no leakage nor abnormal findings.

## Discussion

Foreign body (FB) ingestion is a common problem in children and can cause serious complications (1). In adults, foreign body swallowing happens commonly when eating food and usually consists of non-bony food bolus or the bone of fish or chicken (2). Predisposing factors such as stenosis, malignancies, esophagus rings, and achalasia may facilitate a foreign body remaining in the upper gastrointestinal tract (3). In people of older ages, dentures are commonly swallowed (4). Decreased strength of the senses in the throat and oral cavity due to senility and the increasing population of denture users lead to increased numbers of denture swallowing cases. Foreign body ingestion may occur after trauma, sleep, decreased consciousness, or poisoning (5), and symptoms such as nausea, vomiting, dysphagia, odynophagia, tenderness of the neck, sore throat, secretion of saliva, and regurgitation of undigested food may be present (4, 6). These symptoms are more significant if ingestion occurs more acutely (6). In cases in which the foreign body remains for a longer time, systemic symptoms such as fever and respiratory symptoms such as cough, apnea, and stridor may also be noted (7). The interesting point of this case is that the patient had a foreign body remain in his esophagus for about nine years according to his medical history, yet he had no sign except dysphagia beginning 5 years prior to his referral to the clinic. A foreign body remaining in the esophagus is not uncommon, but remaining for a duration of longer than 6 months in addition to no symptoms associated with it is a rare condition (8). Following foreign body ingestion, patients usually present with dysphagia (92%) and tenderness of the neck (60%). Other symptoms include the inability to swallow oral secretions, throat pain, painful swallowing, hyper-salivation, retrosternal fullness, and regurgitation of undigested food (9, 10). According to studies, the complications of chronically retained foreign body in the esophagus are less likely to be present in children than in adults, and children show fewer symptoms. Chronically retained foreign bodies may be associated with complications such as aortoesophageal fistula, tracheoesophageal fistula, laceration, perforation, respiratory symptoms, and esophageal diverticulum; thus, the foreign body should be extracted promptly following diagnosis, especially in the elderly (11). To select the proper technique for extracting the foreign body, its size, shape, stiffness, and position in the esophagus should be noted. Esophagoscopy is known to be a safe, fast, and acceptable method in most cases, but complications such as rupture of the esophagus or aortic or pericardial membrane, extensive bleeding, or retrosternal abscess might also occur (12, 13). It is advisable to consider using esophagotomy in complicated cases.

## Conflict of interests

The authors declare that they have no conflict of interest.
